# Molecular architecture of the Ub-PCNA/Pol η complex bound to DNA

**DOI:** 10.1038/srep15759

**Published:** 2015-10-27

**Authors:** Wilson C. Y. Lau, Yinyin Li, Qinfen Zhang, Michael S. Y. Huen

**Affiliations:** 1 School of Biomedical Sciences, The University of Hong Kong, Hong Kong, China; 2 State Key Laboratory of Brain and Cognitive Sciences, The University of Hong Kong, Hong Kong, China; 3 State Key Laboratory of Biocontrol, School of Life Sciences, Sun Yat-sen University, Guangzhou 510275, China

## Abstract

Translesion synthesis (TLS) is the mechanism by which DNA polymerases replicate through unrepaired DNA lesions. TLS is activated by monoubiquitination of the homotrimeric proliferating cell nuclear antigen (PCNA) at lysine-164, followed by the switch from replicative to specialized polymerases at DNA damage sites. Pol η belongs to the Y-Family of specialized polymerases that can efficiently bypass UV-induced lesions. Like other members of the Y-Family polymerases, its recruitment to the damaged sites is mediated by the interaction with monoubiquitinated PCNA (Ub-PCNA) *via* its ubiquitin-binding domain and non-canonical PCNA-interacting motif in the C-terminal region. The structural determinants underlying the direct recognition of Ub-PCNA by Pol η, or Y-Family polymerases in general, remain largely unknown. Here we report a structure of the Ub-PCNA/Pol η complex bound to DNA determined by single-particle electron microscopy (EM). The overall obtained structure resembles that of the editing PCNA/PolB complex. Analysis of the map revealed the conformation of ubiquitin that binds the C-terminal domain of Pol η. Our present study suggests that the Ub-PCNA/Pol η interaction requires the formation of a structured binding interface, which is dictated by the inherent flexibility of Ub-PCNA.

The integrity of the genome is constantly challenged by endogenous and exogenous DNA-damage agents^1^,^2^. To minimize the deleterious effects of DNA damage on gene expression and to ensure accurate inheritance of genetic information, cells have evolved various DNA-damage tolerance (DDT) and repair pathways to maintain genome stability^2^,^3^,^4^. DNA lesions leading to prolonged stalling of replication forks are either avoided by homology-dependent template switching or bypassed by TLS, both of which are DDT pathways crucial to allow replication to proceed during the S and G2 phases of the cell cycle without repair^3^,^5^,^6^. TLS involves the temporary replacement of the replicative polymerase by specialized, low-fidelity polymerases to replicate through damage bases or bulky adducts on DNA at the cost of increased mutation rates^5^. The Y-family polymerases are generally involved in nucleotide insertion directly opposite the lesions^7^. Human encodes four Y-Family polymerases (Pol η, Pol ι, Pol κ and REV1); each has its own specificity for the template base and the incoming nucleotide^8^,^9^. For example, Pol η can efficiently bypass UV-induced cyclobutane pyrimidine dimers (CPDs) and cisplatin-induced intrastrand crosslinks^8^,^10^,^11^,^12^, while Pol κ can efficiently bypass Benzo[a]pyrene-adducted guanines^13^. Pol η is by far the best-studied polymerase of the Y-Family whose inactivation causes xeroderma pigmentosum variant, a genetic disease characterized by severe sensitivity to sunlight and predisposition to skin cancer^14^,^15^. Reduced expression of Pol η, ι and κ has also been correlated with the development of lung, stomach and colorectal cancers^16^. Moreover, TLS has been implicated in the repair of interstrand DNA crosslinks, further highlighting the fundamental role of TLS polymerases in genome stability maintenance^4^,^17^,^18^.

TLS is activated by the monoubiquitination of PCNA at lysine-164 in response to replication blockage^19^. PCNA is a DNA sliding clamp with a pivotal role in replication^20^. Not only does it regulate the activities of both replicative and TLS polymerases, it also acts as a scaffold to recruit various cellular proteins involved in DNA repair and cell cycle regulation. The Rad6-Rad18 ubiquitin conjugating/ligase complex mediates the monoubiquitination of PCNA, which in turn recruits TLS polymerases to the sites of DNA lesions^19^,^21^. Most PCNA-binding partners harbor a short sequence motif called the PCNA-interacting protein box (PIP-box) that can fit into a cavity on the surface of PCNA^22^. A non-canonical PIP-box has also been identified among Y-Family polymerases with the exception of REV1^23^, which was shown to interact with PCNA *via* a BRCT domain^24^. All Y-Family polymerases bind the Ub-PCNA with higher affinity than unmodified PCNA, owing to the presence of at least one ubiquitin-binding domain (UBD) at the C-terminal regions of these enzymes^23^,^25^,^26^. The fact that the non-canonical PIP-box has lower affinity for PCNA than a canonical PIP-box such as the one found in the replicative polymerase is consistent with the evidence that Y-Family polymerases are only recruited to the DNA damaged sites upon monoubiquitination of PCNA by Rad6-Rad18.

Crystal structures have been determined for the N-terminal catalytic core of Pol η in complex with normal and various CPD-containing oligonucleotides^27^,^28^. The molecular architecture of the catalytic core shares homology with other Y-Family polymerases and is comprised of the palm, finger, thumb and little finger domains^7^,^8^,^29^. Pertinent to its role in DNA damage bypass, the enlarged active site in the palm domain can readily accommodate the crosslinked thymine dimer^27^,^28^. Pol η also acts as a molecular splint to maintain the damaged, distorted DNA in its normal B-form configuration by forming extensive interactions with the template backbone^27^. On the other hand, the C-terminal, non-catalytic domain of Pol η contains multiple motifs and regions including the PIP-box and the UBD (called UBZ domain in Pol η) as mentioned above for interactions with partner proteins^7^,^26^. This region was predicted to be mostly unstructured based on bioinformatics analysis but may undergo disordered-to-ordered transition upon binding to its cognate partner proteins^30^.

The structure and dynamics of Ub-PCNA have been probed using biophysical methods and computational modeling^31^,^32^,^33^,^34^,^35^. Despite being intrinsically flexible, the conjugated ubiquitins were found to occupy several major, discrete positions on PCNA^34^. Having the propensity to sample discrete positions on PCNA was suggested to be important for the specific recognition of its partner proteins such as Pol η^33^,^34^,^35^. However, the exact biological implication for these discrete positions has remained enigmatic. As a first step towards understanding the Ub-PCNA/Pol η interaction, we used EM of single particles to determine the three-dimensional (3D) structure of the native Ub-PCNA in complex with full-length Pol η and DNA. Our structural data unveil the distinct ubiquitin conformation recognized by Pol η and indicate that intrinsic flexibility of the ubiquitin on PCNA provides the accessible surface for its binding partners.

## Results

### Overall structure of the ternary complex and molecular docking

**Figure 1:**
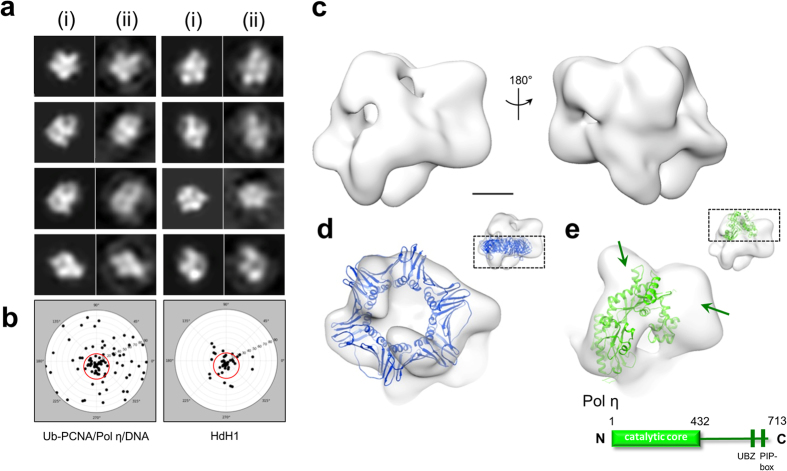
Map validation, overall structure of the 3D reconstruction and atomic model docking. (**a**) Comparison between representative map projections (i) and the corresponding reference-free class averages (ii) in the same orientation to demonstrate self-consistency of the final map. (**b**) Tilt-pair test to validate the overall accuracy of the map and its absolute hand. Pairs of images were acquired at 0° and +10° for the Ub-PCNA/Pol η/DNA ternary complex and as control for *Haliotis diversicolor* hemocyanin isoform 1 (HdH1). The input model for the control tilt-pair test was created by low-pass filtering the previously published 4.5 Å resolution map (EMDB ID 5585) to 20 Å resolution. (**c**) Surface views of the final map. (**d**) The crystal structure of human PCNA (PDB ID 1VYM) was fitted to the bottom layer of the map. The scale bar represents 25 Å. (**e**) The crystal structure of the N-terminal catalytic core of Pol η (PDB ID 3MR2) was fitted to the top layer of the map. Unoccupied density corresponding to the C-terminal domain of Pol η is indicated with dark green arrows.

To reconstitute the Ub-PCNA/Pol η/DNA complex from its purified components, we employed the previously published *in vitro* enzymatic method to produce Ub-PCNA bearing a native isopeptide bond on Lys-164 using a S22R point mutant of the promiscuous E2 UbcH5c, which specifically catalyzes PCNA monoubiquitination without formation of ubiquitin chains^33^. Following the purification of Ub-PCNA and Pol η, a binding assay was carried out to confirm their assembly onto a DNA template-primer substrate (Supplementary Fig. S1). The resulting complex assembled onto DNA yielded monodisperse particles on negatively stain grids that were amenable for single-particle analysis when examined by EM. Because of the relatively small size of the complex (~200 kDa), we pursued structural analysis with negative-stain EM. A final 3D map was determined to a resolution of 22 Å by the gold-standard refinement with two independent starting models (Supplementary Fig. S2). Besides the excellent match between the projections of the map and the reference-free class averages (Fig. 1a), the tilt-pair validation provided support to the accuracy of the final reconstruction (Fig. 1b).

The EM map revealed an overall two-tiered architecture similar to that of the PCNA-family B polymerase (PolB)-DNA complex from archaea (Fig. 1c)^36^. The crystal structure of the PCNA, which forms a toroidal trimer with pseudo six-fold symmetry, could be unambiguously fitted into the map in a distinct location (Fig. 1d). PCNA instead of Ub-PCNA was used initially for fitting, considering the multiple conformations of the conjugated ubiquitins on PCNA adopted in existing atomic structures^31^,^32^. Cross-correlation coefficient was used to distinguish the current fit from the one obtained by 60° rotation of the crystal structure of PCNA along its pseudo six-fold axis. The reliability of the fit was further supported by the agreement between the identified positions of the ubiquitins from the map (described below) and their expected positions when covalently attached to PCNA in the current orientation through the Lys-164 linkage. Inferred from the published model of the PCNA-PolB-DNA complex^36^ (Supplementary Fig. S3), the large remaining portion of the map above the PCNA ring was assigned to Pol η. The catalytic core of human Pol η (1–432) has been previously crystallized in complex with various DNA substrates^27^, while the C-terminal domain has been predicted to be mostly disordered except for the regions consisting of the UBZ domain (628–662) and the PIP-box (702–708) that are responsible for interacting with ubiquitin and PCNA, respectively^23^,^30^,^37^. Additionally, two REV1-interacting regions, a nuclear localization signal and a second putative PIP-box immediately downstream of the catalytic core, are also found within this region^7^,^26^. In attempt to locate the catalytic core in the Pol η density, we identified the elongated mass being a density that could accommodate the palm, finger and the little finger domain altogether. Consequently, this constraint allowed a unique orientation for fitting the crystal structure of catalytic core into the EM map (Fig. 1e). The docking analysis suggested that the unoccupied densities in the upper portion of the map should represent the C-terminal domain of Pol η (Fig. 1d). This region of unoccupied densities accounts for ~40% of the total volume of the Pol η subunit, in agreement with the relative molecular mass of the C-terminal domain as compared to the full-length Pol η (282 out of 713 amino acids).

### Localization of the DNA

**Figure 2:**
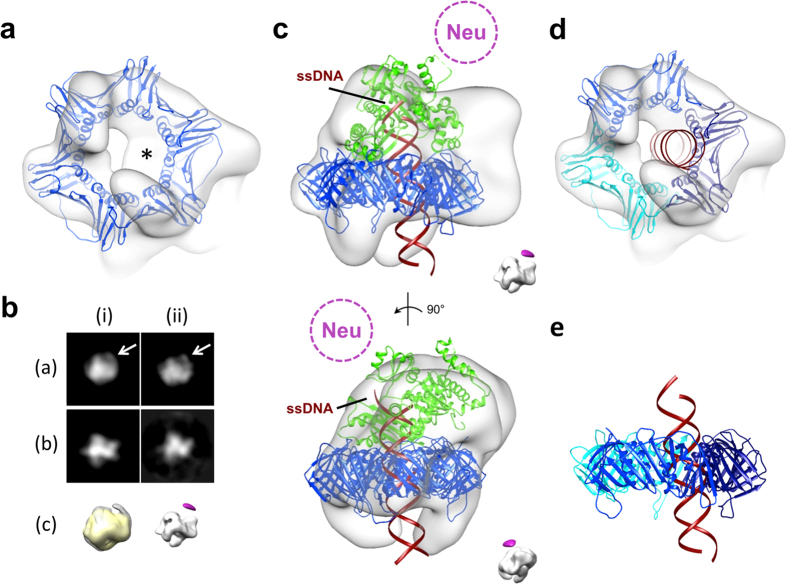
Localization of the DNA and its interaction with the complex. (**a**) Viewed from the bottom, the map is shown with the crystal structure of PCNA (PDB ID 1VYM) fitted in. The map is displayed at a slightly higher threshold to reveal that the central hole of PCNA is partially occupied with an extra density (indicated by an asterisk). (**b**) Map projections (i) and reference-based class averages (ii) showing the presence of a prominent density (white arrow) in the neutravidin (neu)-labeled map (**a**) but absence in the unlabeled map (**b**) in 2D. 3D map of the neu-labeled complex (mesh) is overlaid onto the unlabeled map filtered to the same resolution (yellow surface; c; left). The unlabeled map showed no equivalent density even if the map was displayed at significantly lower thresholds. A difference map (purple surface) calculated between the labeled and the unlabeled map is shown along with the unlabeled map to depict the position of neu relative to the complex (c; right). Orientation of the maps correspond to the same views as in (**a**,**b**). (**c**) The map is shown with the available crystal structures fitted in. The catalytic core of Pol η is in green and PCNA in blue. The approximate position for neu is indicated. A 25-mer DNA duplex was modeled into the reconstruction with the template ssDNA terminus on the polymerase side to match with the location of the neu density. Insert shows the relative location of the experimental neu density calculated from difference mapping. (**d**) Same view as in (**a**). The extra density in the central hole of PCNA can be attributed to the DNA. Each of the PCNA protomers is distinguished by a different colour to underscore the specific interaction between DNA and one of the PCNA subunits. (**e**) A side view of the PCNA crystal structure is shown with the DNA penetrating through its central hole. A noticeable tilt (~13°) is observed between the DNA and the PCNA ring. The colour code for the PCNA ring is the same as in (**d**).

Consistent with the presence of DNA in the complex, the central hole of PCNA is partially occluded by a conspicuous density, reminiscent of the DNA passing through the ring (Figs 1d and 2a). Importantly, this density adheres asymmetrically to the inside of the PCNA ring when the map is rendered at a slightly higher density threshold, implying a non-uniform interaction of the DNA with the PCNA ring. However, our reconstruction could not resolve the entire DNA strand spanning the complex perhaps due to the flexibility of the bound DNA, and/or binding of heavy metal salts in the DNA grooves resulting in the general difficulty in visualizing DNA in negative stain as noted in previous studies^36^,^38^,^39^,^40^,^41^. While the interaction of DNA with the rest of the complex had been confirmed by crosslinking (Supplementary Fig. S1), we sought to directly visualize and determine the polarity of the DNA in the complex by localizing the terminus of the single-stranded region of the template-primer substrate using EM. To this end, a one-end biotinylated DNA template-primer duplex was generated and complexed with Ub-PCNA/Pol η, and following the addition of neutravidin, the resulting complexes were imaged as before. Comparison of the 2D class averages and 3D reconstructions determined for the labeled and unlabeled complex revealed an extra mass of density that was present only in the labeled map (Fig. 2b). The unlabeled map showed no equivalent density even if the map was displayed at lower thresholds. The appearance of this extra density could be attributed to the attachment of neutravidin to the biotinylated terminus of the single-stranded DNA (ssDNA) region. Its position relative to the complex is in accord with the single-stranded region of the template strand bound to the side of the polymerase, as would be expected if the DNA was assembled in the biologically relevant orientation within the complex (Fig. 2c). The fact that this extra density appears smaller than its anticipated mass reflects the intrinsically mobile nature of the ssDNA, leading to variability in the position of the bound neutravidin at the periphery of the complex (Fig. 2b,c). The labeling approach not only substantiated the conclusion that the ternary complex contained DNA, but also provided strong support to the fitted models.

Since the DNA density was not clearly resolved in our reconstruction, we modeled the primed DNA into the molecular model to gain insights into its interaction with the rest of the complex. Intriguingly, our model suggests that the DNA backbone interacts asymmetrically with the central hole of PCNA, only forming stable contact with one of the PCNA monomers (Fig. 2d). In accord with the EM map, the position of the penetrating DNA coincides with the density found in the inside of the PCNA ring. Furthermore, the DNA transverses the plane of the PCNA ring at an oblique angle of ~13° (Fig. 2e).

### Conformations of conjugated ubiquitins on PCNA

**Figure 3:**
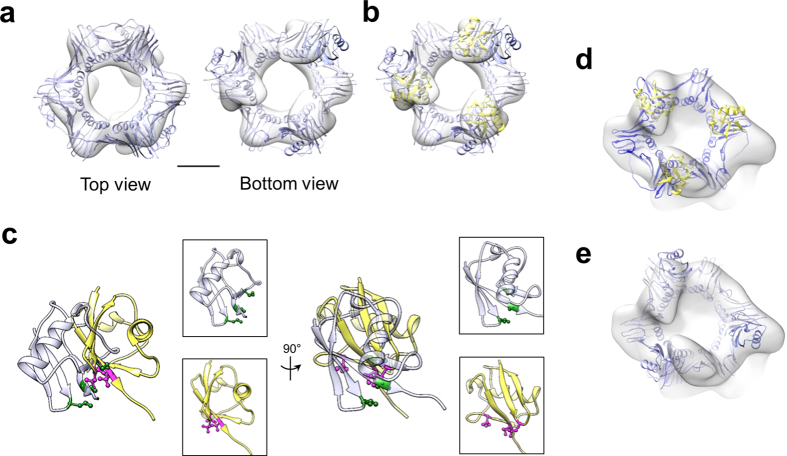
Structure of the native Ub-PCNA exhibits a conformation different from that of the Ub-PCNA bound to Pol η and DNA. (**a**) Top and bottom views of 3D reconstruction of with the fitted split-fusion Ub-PCNA crystal structure (light blue; PDB ID 3L0W). Scale bar represents 25 Å. (**b**) Bottom view of (**a**) but with ubiquitin (yellow; PDB ID 1UBQ) independently fitted into the map. The different orientation of the ubiquitins observed between the two structures suggests local flexibility. (**c**) Close-up views of the two ubiquitins overlaid. The orientation of the ubiquitin (yellow) fitted according to the native Ub-PCNA EM map exposes the hydrophobic residues (magenta; Leu8, Ile44, Val70) necessary for its interaction with UBD. The same hydrophobic residues on ubiquitin (semi-transparent light blue) in the split-fusion crystal structure are colored green. Left, end-on view towards to the central symmetry axis of PCNA; Right, 90° rotation of the end-on view. (**d**) EM map of the ternary complex with the fitted ubiquitin orientations from (**b**) and PCNA (PDB ID 1VYM) (**e**) EM map of the ternary complex with the fitted split-fusion PCNA crystal structure.

We next analyzed the remaining densities associated with the PCNA ring in the 3D reconstruction. The configuration of the two lobes of densities that are attached to PCNA highly resembles the crystallographic positions adopted by the ubiquitin moieties on the back face of the split-fusion Ub-PCNA structure^32^, whereas the third lobe appears to reside to the side of the PCNA ring. On the basis of this observation, we assigned the lobes to the monoubiquitins conjugated to PCNA. In parallel, we calculated a 3D reconstruction of the Ub-PCNA to 17 Å resolution (Fig. 3a and Supplementary Fig. S2). While the resulting map allowed the crystal structure of PCNA to be fitted into the ring portion with high fidelity, attempt to fit in the intact split-fusion Ub-PCNA structure as a rigid body into the EM map required reorientation of the ubiquitin moieties relative to the corresponding EM densities. This disparity can be explained by the conformational flexibility of the ubiquitins, a well-known property of the Ub-PCNA complex^33^,^34^,^35^. The ubiquitin densities are better resolved in the Ub-PCNA map compared to those in the map of the ternary complex, allowing the crystal structure of ubiquitin to be fitted reliably into the densities (Fig. 3b). Notably, the ubiquitin is rotated to an orientation that exposes the canonical hydrophobic surface responsible for interacting with the UBZ domain of Pol η and other proteins (Fig. 3c)^32^,^37^. Even though this conformation is likely populated by specific interactions of the complex with the stain and/or the carbon support, our analysis suggests the existence of local flexibility of the ubiquitin. This is line with the observation that the ubiquitin must undergo rotation from the crystallographic position in the split-fusion structure in order to release the association of its canonical hydrophobic surface from PCNA, thereby allowing for interaction with other partner proteins *via* the same surface^32^. Interestingly, this upright orientation exhibited by the ubiquitins in the map of Ub-PCNA matches closely the orientation of the two ubiquitins localized to the back face of PCNA in the ternary complex (Fig. 3d), yet both of which are different from the one observed in the split-fusion Ub-PCNA structure (Fig. 3d,e).

**Figure 4:**
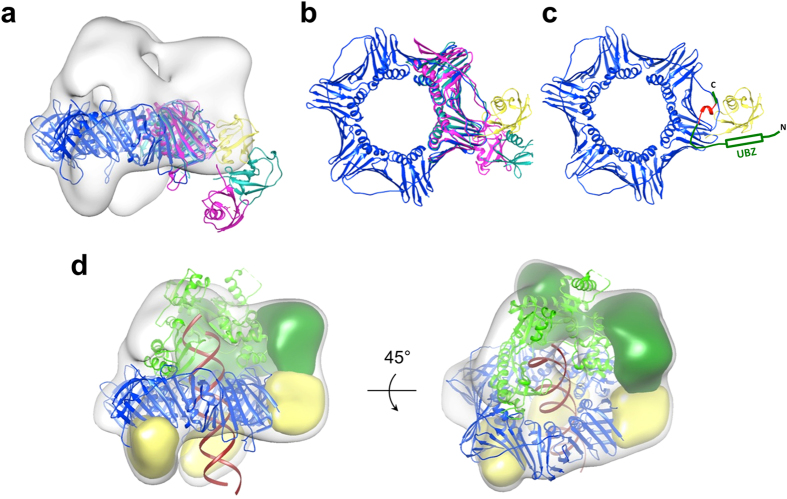
The ubiquitin adopts the ‘flipped-out’ conformation to interact with the C-terminal domain of Pol η. (**a**) The protomer of the native Ub-PCNA crystal structure in the extended conformation (light sea green; PDB ID 3TBL) and the SUMO_K164_-PCNA^mono^ (magenta; PDB ID 3V61) are fitted into the EM map of the ternary complex. The crystal structure of the ubiquitin (yellow; PDB ID 1UBQ) is manually docked into the map to show its approximate position relative to the other crystal structures. (**b**) Top view of (**a**) with the EM map omitted for clarity. (**c**) The crystal structure of the PCNA bound to a 20-mer peptide carrying PIP-box of Pol η (PDB ID 2ZVK) is fitted to the same orientation within the EM map as in (**a**). Viewing direction is same as in (**b**). The PIP-box sequences are colored red. The proposed interaction between the UBZ of Pol η with ubiquitin is depicted. (**d**) Schematic representation of the overall ternary complex in two different orientations showing the interaction of the C-terminal domain of Pol η with only one of the conjugated ubiquitins on PCNA. Map segments corresponding to the C-terminal domain of Pol η and ubiquitin are colored dark green and yellow, respectively.

In the structure of the ternary complex, one of the ubiquitins undergoes a dramatic rearrangement with respect to the PCNA ring and swings out almost 90° away from its symmetry axis, forming contact with the C-terminal domain of Pol η (Fig. 4a). This large motion is almost certainly induced by the binding of the ubiquitin to the UBZ domain of Pol η. Overlay of the native Ub-PCNA crystal structure with our EM map suggests that the third ubiquitin occupies an intermediate position relative to the ubiquitin seen in the extended structure of the native Ub-PCNA as it projects radially away from the PCNA ring (Fig. 4a). Viewed from the front face of PCNA, the third ubiquitin also shifts to the right of the ubiquitin derived from the crystal structure (Fig. 4b).

## Discussion

Monoubiquitination of PCNA represents the key signal for the switch between high-fidelity and specialized DNA polymerases to bypass a variety of DNA lesions at stalled replication sites. This current work provides a structural basis for understanding the mechanism underlying the direct recognition of Ub-PCNA by the TLS polymerase Pol η, and highlights the intrinsic flexibility of Ub-PCNA as a requirement for the Ub-PCNA/Pol η interaction. Moreover, we anticipate that the mechanism defined here will be broadly applicable to other Y-Family polymerases, given that they all possess ubiquitin-binding domains along with a PCNA-binding domain.

A molecular model of the Ub-PCNA/Pol η/DNA ternary complex was constructed by rigid-body fitting of crystal structures into the EM map. The fitting also identified regions of the density map that corresponded to the C-terminal domain of Pol η, atomic structure of which is unavailable. Although it is predicted to be mostly unstructured in the absence of its binding partner, the C-terminal domain likely acquires an ordered structure upon binding to Ub-PCNA, as suggested by the good agreement between calculated and experimental molecular masses determined for this domain. In contrast, neutravidin showed a decrease in volume density in the reconstruction when tethered to the complex *via* a flexible ssDNA region.

On the basis of neutravidin labeling experiment and the fact that Pol η interacts with the front face of the PCNA, the structure presented here should represent the complex in the polymerizing mode. Previous work proposed models for the PCNA-PolB interaction in the polymerizing and the editing mode^36^. With the catalytic domain of the Pol η sitting atop PCNA, our structure, however, concurs with the structure of the PCNA/PolB/DNA complex predicted for the editing mode (Supplementary Fig. S3) but not the polymerizing mode, where the replicative polymerase PolB would be expected to rise up. The relative orientation of the DNA bound to the replicative polymerase *via* the polymerase and exonuclease domain gave rise to the observed difference in conformation between the polymerizing and editing mode of the complex, respectively^36^,^42^. By contrast, Pol η, a Y-Family polymerase, has a very different structure and lacks the exonuclease domain (Supplementary Fig. S3). In our model, the DNA substrate makes a notable asymmetric interaction with the PCNA ring, with the relative tilt of the DNA with respect to the PCNA ring identical to that observed in the editing PCNA-PolB complex, which is ~13°^ ^36. The tilted orientation of various angles of the DNA and its asymmetric interaction with PCNA has been predicted by molecular dynamics simulations and demonstrated experimentally in a number of previous studies^38^,^43^,^44^,^45^,^46^. In fact, the proposed switch between the polymerizing and editing mode of replicative polymerases entails tilting of the DNA through the PCNA ring by up to 40°^ ^42. A tilted DNA was also observed in the 9-1-1/FEN1/DNA complex, although the mode of interaction of the DNA was different for the 9-1-1 versus PCNA, with 9-1-1 forming stable contacts with the DNA *via* residues distributed evenly among its three subunits^38^. Together, our data strengthens the current view by which the diverse but specific interactions of the DNA with the sliding clamps are critical to the recruitment of distinct protein partners. It is tempting to speculate that the ability of the DNA to tilt through the clamp rings may facilitate not only the switch between the polymerizing and editing modes of replicative polymerases, but also between replicative and TLS polymerases. Of note, the phenomenon of DNA switching among multiple partners simultaneously bound to same sliding clamp has already been proposed for both PCNA and the prokaryotic β clamp^43^,^44^.

The flexibility inherent in ubiquitin- and ubiquitin-like protein-conjugated substrates has been implicated in both the recognition and selection of their cognate protein partners. In the case of Ub-and SUMO-PCNA, recent analyses using small-angle X-ray scattering along with hybrid modeling methods have revealed a different degree of flexibility among them^34^. The ubiquitin conjugated on PCNA at K164 was found to occupy three major, discrete positions on the PCNA surface; two of which were congruent with the structures determined for native and the split-fusion Ub-PCNA by X-ray crystallography, where the ubiquitin resides on the back face or projects radially away from the PCNA ring, respectively. The biological significance of these identified positions has thus far remained elusive. SUMO, on the other hand, was found to occupy extended positions by simple tethering to PCNA and conferred a much greater degree of flexibility in solution compared to Ub-PCNA, consistent with the variability in SUMO orientation among different conformers of SUMO-PCNA in the Srs2-SUMO-PCNA crystals^47^. Interpreting our map in light of these findings suggests that the ubiquitin moiety, which associates with Pol η, adopts the flexibly ‘flipped-out’ conformation, albeit less extended radially than the one seen in the native Ub-PCNA crystal structure. The conformation of the crystallographic conformer of SUMO_K164_-PCNA^mono^, wherein SUMO makes no substantial contacts to PCNA^47^, also resembles to the conformations adopted by either ubiquitins in the EM and the crystal structure, although to a lesser extent as SUMO occupies a position most towards the back face of PCNA (Fig. 4a,b). The observation that Pol η binds and stabilizes the ubiquitin in the ‘flipped-out’ conformation is striking, and strongly indicates the formation of a composite surface between ubiquitin and PCNA for Pol η binding, in agreement with the structured interface model^34^. With a distance of only 41 residues apart, juxtaposition of the PIP-box and the UBZ domain likely allows Pol η to simultaneously engage both PCNA and ubiquitin on a single protomer subunit of the PCNA ring. The current map also argues against the importance of the third position identified for the conjugated ubiquitin on PCNA at K164 in the context of TLS, which involves the anchoring of ubiquitin to the side of the PCNA ring directly adjacent to the subunit-subunit interface^35^. An alternative set of conformations have been identified for ubiquitin conjugated at K107 on the yeast PCNA that did not include the ‘flipped-out’ conformation as in the case for the K164 conjugate^48^. Consistent with the notion that the ‘flipped-out’ conformation is essential for the recognition of Pol η, in yeast, modification of K107 of PCNA has been implicated in the activation of a DDR pathway in response to DNA ligase I deficiency that is independent of TLS. This pathway also appears to be conserved in humans, although ubiquitination likely occurs at K110 instead.

The structure of the ternary complex presented here lead to a conclusive proof of the requirement for monoubiqitination of a single PCNA protomer in the direct association of Pol η during DNA damage bypass. The detection of UV-induced Pol η foci in cells expressing PCNA heterotrimers of WT and K164R mutant subunits also suggested that triple monoubiquitination was not a prerequisite for Pol η-mediated TLS^49^, in line with Pol η carrying only one Ub-binding motif at its C-terminus. With only one of the three ubiquitins bound to Pol η, the remaining two unoccupied ubiquitins are potentially free to interact with other binding partners. Even though the ubiquitins on the back face of PCNA were found to interact with PCNA in the crystal *via* the canonical hydrophobic surface^32^, we showed that they could reorient themselves to expose this same surface for UBDs in solution due to the weak interaction between ubiquitin and PCNA^32^. Together, our data is consistent with Ub-PCNA functioning as a tool belt to recruit enzymes to the back face when needed, and is compatible with the two-polymerase mechanism^50^ and a recently discovered Pol η-independent DDR pathway that is activated by co-modification of multiple PCNA protomers by ubiquitins^49^.

In summary, we report for the first time, to our knowledge, the structure of a full-length TLS polymerase/Ub-PCNA complex bound to DNA. The structure mapped the location of the C-terminal domain of Pol η, elucidated the mode of interaction of the DNA with the complex, and captured the conformation of the ubiquitin competent for binding to Pol η. A more detailed molecular view awaits high-resolution structure determination by cryo-EM with direct electron detection^51^.

## Methods

### Protein production

Human PCNA with an N-terminal His_6_-tag was overexpressed in BL21(DE3) cells. Cells were harvested and resuspended in buffer A (50 mM Tris-HCl, 300 mM NaCl, 20 mM imidazole, 10% glycerol (v/v), 0.001% (v/v) PMSF, pH 8.0). After sonication and centrifugation, the supernatant was loaded onto a 1-mL HisPur Ni-NTA column (Thermo Scientific). The column was washed with 10 column volumes (CVs) of buffer A followed by 10 CVs of buffer A containing 40 mM imidazole. Proteins were eluted stepwise with 300 mM imidazole in buffer A. Pooled fractions containing PCNA were concentrated and buffer exchanged into 20 mM HEPES, 150 mM NaCl, pH 7.5, using an Amicon Ultra 30 K centrifugation unit (Millipore). Monoubiquitination of PCNA was performed in low salt buffer and under alkaline conditions, as described previously^33^, in the presence of 80 nM E1 (Boston Biochem), 32 μM Flag-ubiquitin (Boston Biochem), and various concentrations of PCNA to UbcH5c (S22R) at 1:10 molar ratio. The reaction was performed for 30 min at 37 °C in a buffer containing 50 mM malic acid-MES-Tris, 25 mM NaCl, 3 mM MgCl_2_, 0.5 mM TCEP, 3 mM ATP, pH 9.0. The resulting Ub-PCNA was purified from the reaction mixture using M2 Affinity agarose gel (Sigma-Aldrich) and excess Flag-Ub was subsequently removed using an Amicon Ultra 30 K centrifugation unit (Millipore). To obtain soluble, full-length human Pol η in a bacterial system, the protein was expressed at low basal level without induction from a low-copy-number vector (pJM879) in an *E. coli* strain lacking three endogenous polymerases (RW644)^52^,^53^. The expressed Pol η, which harbored an N-terminal His_6_-tag, was purified *via* metal chelate affinity column as described with modifications^52^. Briefly, harvested cells were resuspended and sonicated in buffer A with the addition of Roche Complete protease inhibitor cocktail (EDTA-free) and 10 mM beta-mercaptoethanol (BME). The lysate was clarified by centrifugation prior to applying onto a 1-mL HisPur Ni-NTA column (Thermo Scientific) equilibrated in the same buffer. The column was washed with 3 CVs of buffer W1 (20 mM Tris-HCl, 1 M NaCl, 20 mM imidazole, 10% (v/v) glycerol, 10 mM BME, pH 7.5), followed by 3 CVs of buffer W2 (10 mM sodium phosphate, 300 mM NaCl, 20 mM imidazole, 10% (v/v) glycerol, 10 mM BME, pH 7.7). The protein was finally eluted with 200 mM imidazole in W2 buffer. Fractions containing Pol η were pooled, concentrated and buffer exchanged into 20 mM sodium phosphate, 100 mM NaCl, 10 mM BME, pH 7.3. All purified proteins were flash-frozen and stored at −80 °C until use.

### Specimen preparation and electron microscopy

An equal molar ratio of purified Ub-PCNA and Pol η were mixed and dialyzed against 50 mM Tris-HCl, 5 mM MgCl_2_, pH 8.0, in a Slide-A-Lyzer MINI Dialysis Device (Thermo Scientific) at room temperature for 1 hr. After dialysis, the binary mixture was further incubated with 2.5-fold molar excess of primed DNA at 37 °C for 15 min to form the ternary complex. For the generation of the primed DNA substrate, the complementary synthetic oligonucleotides (49-nucleotide template and 25-nucleotide primer strands) were annealed at 1:1 molar ratio by heating at 95 °C for 5 min followed by gradually cooling to room temperature in a buffer containing 10 mM HEPES, 0.1 mM EDTA, 50 mM NaCl, pH 8.0. The sequences of the oligonucleotides forming the 25/49 DNA substrate are essentially the same as described in^36^. The final Ub-PCNA/Pol η/DNA ternary complex was further stabilized by mildly crosslinking with 0.04% (v/v) glutaldehyde for 10 min at room temperature. The reaction was quenched with 100 mM Tris-HCl and immediately used for EM grid preparation.

Negative stained samples of Ub-PCNA and the ternary complex were prepared using freshly glow-discharged, continuous carbon coated copper grids (Ted Pella). Four microliters of sample at a concentration of 0.1 mg/ml were adsorbed onto the grids for 2 min. The grids were floated sequentially onto a 20 μl drop of distilled water, followed by two 20 μl drops of 2% (w/v) uranyl acetate solution. The stained grids were blotted with filter paper and air-dried. Data was acquired on a JEOL JEM2010 electron microscope operated at 200 kV at a nominal magnification of 50,000× using a defocus range of −0.5 to −2 μm with an electron dose of ~18 e^−^/A^2^. Images were recorded on a Gatan Ultrascan 4 k × 4 k CCD camera with 2.14 Å calibrated pixel size at the specimen level.

### Image processing

All image-processing steps were performed using programs from EMAN2^54^. Particles were selected either manually or semi-automatically using e2boxer.py. The contrast transfer function (CTF) of the particles in each frame average was determined using e2ctf.py. Particles were first phase-flipped in 2D and full CTF correction was performed automatically during the 3D reconstruction step. To remove protein aggregates, contaminants and dissociated complexes, selected particles were subjected to iterative multivariate statistical analysis-based reference-free classification with e2refine2d.py to sort out a homogeneous population of particles for further processing. After sorting, the remaining dataset for the Ub-PCNA/Pol η/DNA ternary complex and Ub-PCNA consists of 7330 and 24413 particle images, respectively. The initial model used for the ternary complex was generated using the e2initialmodel.py subroutine by iterative refinement of 24 reference-free class averages from random blobs. The selection of the initial model from a list of possible pseudo-answers was based on the good agreement between the class averages and the corresponding projections of the model, as well as *a priori* knowledge about the presence of a unique feature in the model, i.e. a hexameric ring structure that corresponds to PCNA. Gold-standard 3D refinement was carried out by projection matching using the SNR-weighted Fourier ring correlation with e2refine_easy.py. For the reconstruction of the Ub-PCNA, a low-pass-filtered map of the native PCNA crystal structure was used as the initial model for iterative 3D refinement with C3 symmetry imposed.

### Localization of the DNA by neutravidin labeling

To localize the ssDNA region thus the orientation of the primed DNA within the complex, a 5′ biotinylated template strand was annealed with the primer strand and the resulting primed DNA substrate was used to form the ternary complex of Ub-PCNA/Pol η/DNA by following the same method as described above for the unlabeled complex. Prior to glutaldehyde crosslinking, neutravidin (1.7 μM) was added into the reaction mixture and incubated for 15 min at room temperature. A total of 3806 particle images were selected for the analysis. Procedures for image processing and 3D reconstruction for the labeled complex were identical to those used for the unlabeled complex, except the final 3D map of unlabeled complex was used as the initial reference.

### Resolution estimation and map validation

The 3D refinement for all reconstructions were carried out by following the gold standard procedure, in which the data were randomly split into two halves and then were refined independently using two starting models with Fourier phases randomized at low resolution^55^,^56^. The resolutions of the reconstructions were estimated from the Fourier Shell Correlation (FSC) at the 0.143 criterion^57^. Comparison between the projections of the 3D map and the corresponding reference-free class averages highlighted the self-consistency of our final structure. For map validation, pairs of images of the same field were recorded at 0 and +10°. The program e2tiltvalidate.py was used to determine the relative orientation of each particle image in the image pairs. Tilt-pair analysis was also carried out for negatively stained *Haliotis diversicolor* molluscan hemocyanin (HdH1) of known handedness under identical conditions to determine the direction of the tilt axis^58^. The formation of a cluster of points centered on the expected tilt axis and around the expected tilt angle on the tilt-pair parameter plot indicated the overall validity of the map and that the map is of correct hand.

### Map interpretation and molecular modeling

The crystal structures of the human native PCNA (PDB ID 1W60) and the catalytic core of the human Pol η (1–432 amino acids) (PDB ID 3MR2) were docked into the EM map using UCSF Chimera^59^. To model the duplex DNA in the B-form conformation passing through the central hole of the PCNA ring, a 25 base-paired DNA with a two-nucleotides overhang was built and then aligned to the original, normal DNA substrate that is in complex with Pol η from the crystal structure (PDB ID 3MR2). The two DNA duplexes matched well except for the region near the active site of Pol η. Computation of the difference map, volume segmentation, visualization and rendering were performed using UCSF Chimera.

## Additional Information

**Accession Numbers:** The E.M. map of Ub-PCNA/Pol η/DNA has been deposited in the EMDB under accession number EMDB-6339. The atomic coordinates for the fitted crystal structures have been deposited in the PDB under accession numbers PDB ID 3JA9 and 3JAA.

**How to cite this article**: Lau, W. C. Y. *et al.* Molecular architecture of the Ub-PCNA/Pol η complex bound to DNA. *Sci. Rep.* **5**, 15759; doi: 10.1038/srep15759 (2015).

## Supplementary Material

Supplementary Information
