# Supplementary material for: Molecular architecture of the Ub-PCNA/Pol η complex bound to DNA
Source: Sci Rep. 2015 Oct 27;5:15759. doi: 10.1038/srep15759 (PMC4621508; doi:10.1038/srep15759)
Supplement: Supplementary Information [file srep15759-s1.pdf]

Molecular architecture of the Ub-PCNA/Pol η complex bound to DNA

Wilson C.Y. Lau[1,*], Yinyin Li[2], Qinfen Zhang[2], Michael S.Y. Huen[1,*]

[1] School of Biomedical Sciences, The University of Hong Kong, Hong Kong, China

[2] State Key Laboratory of Biocontrol, School of Life Sciences, Sun Yat-sen University, Guangzhou 510275, China

*Correspondence: wcylau@hku.hk, huen.michael@hku.hk

Supplementary Figure S1

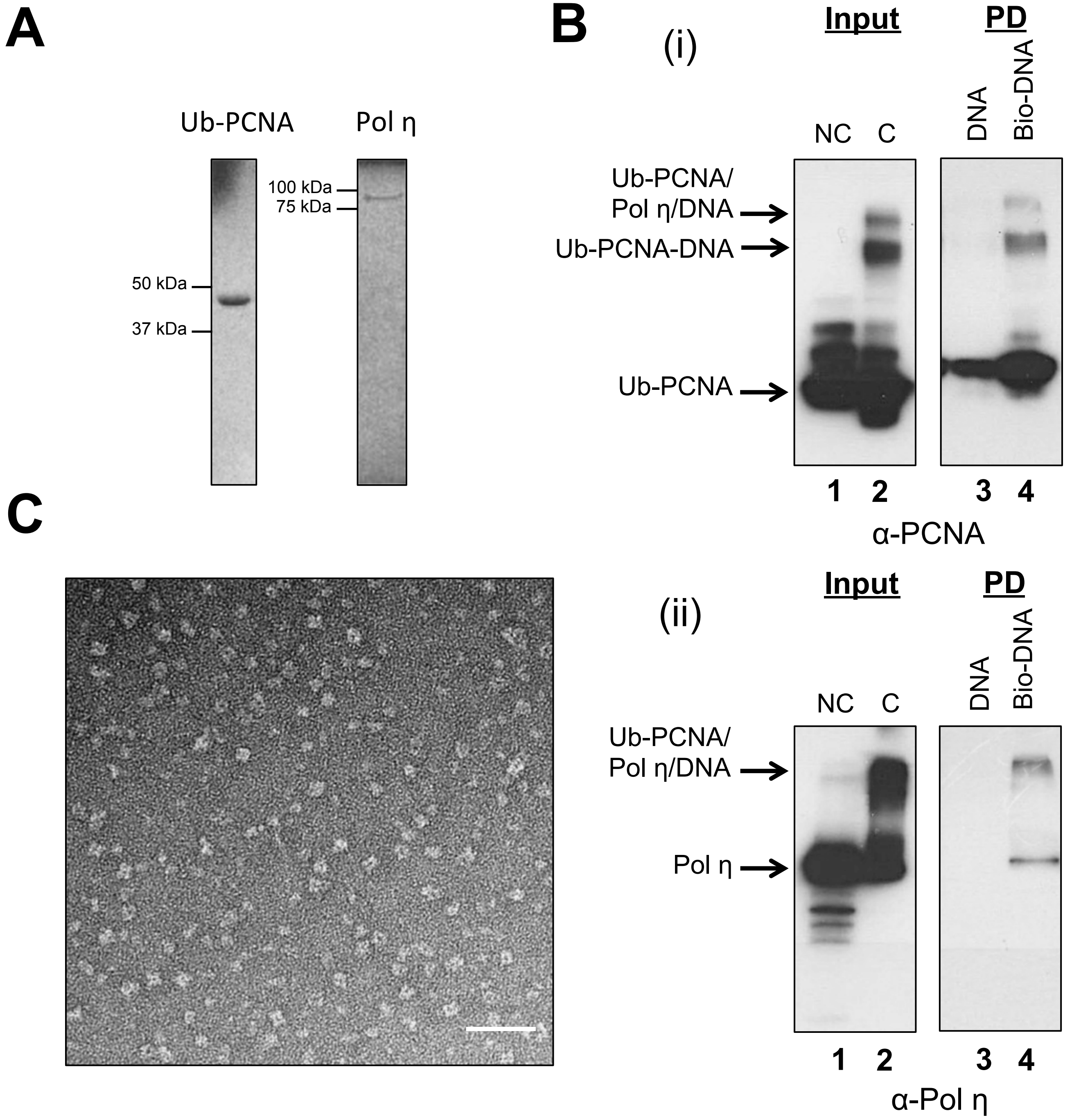

Figure S1. Ub-PCNA, Pol η and DNA can form a ternary complex *in vitro*. (A) SDS-PAGE of the purified proteins. (B) Proteins were incubated with primed DNA with and without a 5' biotin and subjected to glutaldehyde crosslinking. Reaction was stopped and the assembled complexes were pulled down by Strepavidin beads. Crosslinked and non-crosslinked complexes were detected by SDS-PAGE followed by immunoblot analyses using antibodies against (i) anti-PCNA and (ii) anti-Pol η. Lane 1 and 2 shows the input reaction in absence and in the presence of the crosslinker, respectively. Non-biotinylated DNA was used as a negative control for the pull-down reaction (lane 3). Crosslinked binary and ternary complexes (indicated by arrows) were detected in the pull-down (lane 4). (C) A representative EM micrograph of the ternary complex. Scale bar represents 50 nm.

Supplementary Figure S2

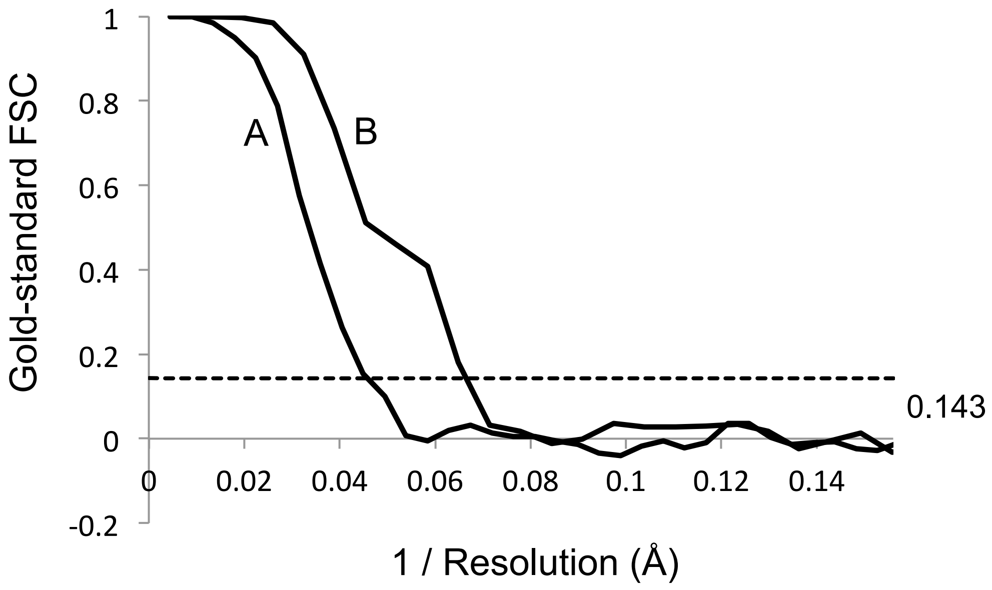

Figure S2. **Gold-standard Fourier shell correlation plots.** Resolution estimation for (A) Ub-PCNA/Pol η/DNA ternary complex and (B) native Ub-PCNA.

Supplementary Figure S3

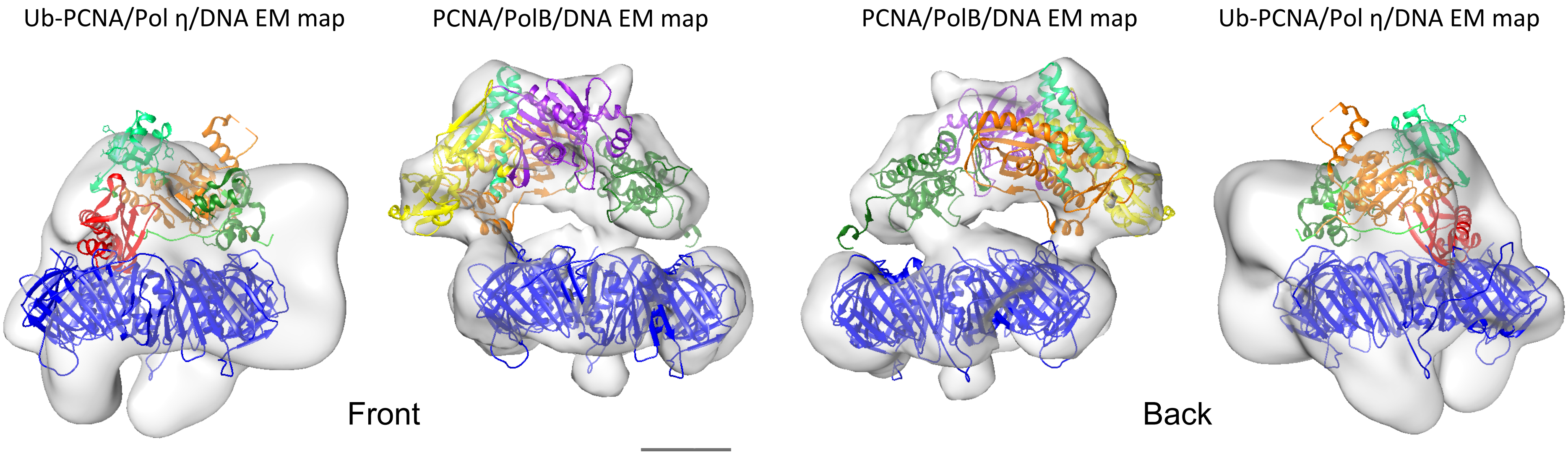

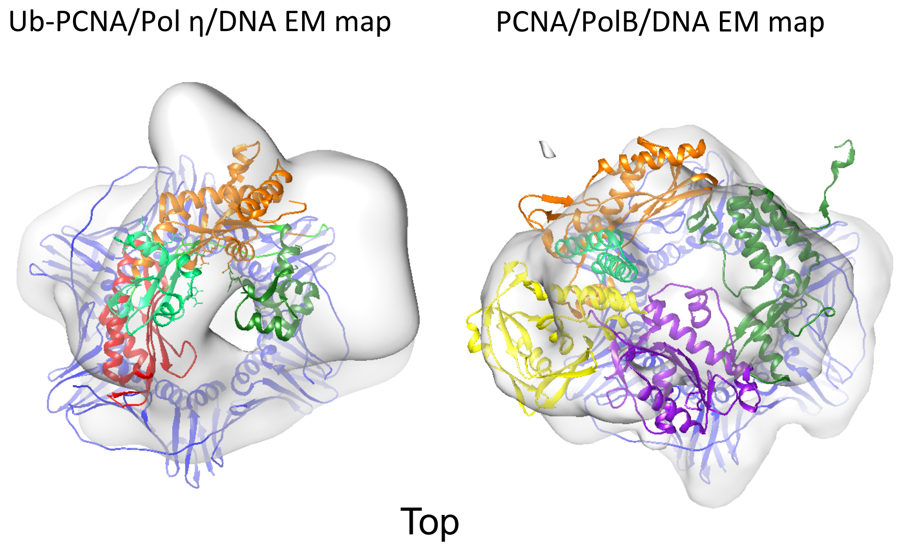

Figure S3. Comparison between the EM structures of Ub-PCNA/Pol η/DNA complex and the PCNA/PolB/DNA complex. The pseudoatomic model of the PCNA/PolB/DNA complex was constructed by docking the family B DNA polymerase from *Pyrococcus furiosus* (PolB) (PDB 3A2F) and PCNA as rigid bodies into the EM map (EMDB 5220). The five domains of PolB are shown in distinct colors (palm, orange; thumb, dark green; fingers, spring green; exonuclease, purple; N-terminal, yellow). The same color scheme also applies to the corresponding domains in Pol η. The little finger domain, which is unique to the Y-family polymerase, is colored red. Note that Pol η does not carry the N-terminal and the exonuclease domain. The scale bar applies to all panels and represents 25 Å
